# A mass spectrometric strategy for absolute quantification of *Plasmodium falciparum *proteins of low abundance

**DOI:** 10.1186/1475-2875-10-315

**Published:** 2011-10-25

**Authors:** Paul M Southworth, John E Hyde, Paul FG Sims

**Affiliations:** 1 Manchester Interdisciplinary Biocentre, Faculty of Life Sciences, University of Manchester, 131 Princess Street, Manchester M1 7DN, UK; 2 Laboratory of Malaria Immunology and Vaccinology, National Institute of Allergy and Infectious Disease, National Institutes of Health, 12735 Twinbrook Parkway, Rockville MD 20814, USA

**Keywords:** Absolute quantification of proteins, enzymes of folate metabolism, heavy isotope labelling, malaria parasites, QconCAT

## Abstract

Selected reaction monitoring mass spectrometry has been combined with the use of an isotopically labelled synthetic protein, made up of proteotypic tryptic peptides selected from parasite proteins of interest. This allows, for the first time, absolute quantification of proteins from *Plasmodium falciparum*. This methodology is demonstrated to be of sufficient sensitivity to quantify, even within whole cell extracts, proteins of low abundance from the folate pathway as well as more abundant "housekeeping" proteins.

## Background

Human malaria caused by *Plasmodium falciparum *is a major global health burden, killing around 1 million people every year [1]. Africa bears the greatest proportion of this burden, with over three-quarters of deaths occurring in African children, accounting for nearly a fifth of all child deaths in sub-Saharan Africa [2]. With the sequencing of the genome [3], as well as great advances in mass spectrometry (MS), characterization of the *P. falciparum *proteome is now technically possible. Information obtained using these techniques should be valuable in informing our future understanding of parasite/human interactions, disease progression and the selection of novel drug targets.

One important recent advance in MS is the development of so-called selected reaction monitoring (SRM). This is an analysis method used in triple quadrupole mass spectrometers. The first quadrupole acts as a mass filter, allowing through only ions of selected mass/charge ratios before they are fragmented, using the second quadrupole as a collision cell. The final quadrupole then acts as a mass filter of resulting fragment ions in a similar way to the first quadrupole, allowing through only fragment ions of a particular, selected mass [4]. Since different peptides of the same mass would be expected to show different fragmentation patterns, this method provides an extra degree of certainty of protein identification compared to MS of the peptides alone.

Further to these advances, recent work from this laboratory [5] has demonstrated that a form of 'soft' extraction of erythrocytic-stage *P. falciparum *parasites can resolve the issue of haemoglobin-derived products, which previously caused great hindrance in downstream methods of protein analysis. In addition, this work demonstrated the utility of the OFFGEL™ (Agilent, UK) isoelectric fractionation system in separating whole *P. falciparum *proteins, rather than tryptic peptides, according to their pI. This fractionation is vitally important in the identification of proteins of low abundance, such as those of the folate and many other metabolic pathways, due to the complexity of the proteome. It is estimated that even in the most comprehensive proteomic studies of the *P. falciparum *life-cycle to date, only ~46% of the predicted gene products were detected [6,7].

As well as identifying proteins of interest by fractionation followed by mass spectrometry, quantification of these proteins is also highly valuable in understanding the dynamics of biological systems. It has been shown a number of times that the expression of a particular gene as measured by mRNA quantification does not necessarily correlate with the level of protein within the cell [8-11]. Moreover, it is known that the *P. falciparum *proteome contains many unstructured proteins that can experience rapid degradation at both mRNA and protein levels [12,13]. In addition, the nature and degree of post-translational modifications, which can determine a number of protein properties including function, localization and activity, can only be analysed at the protein level.

Quantitative proteomics of the *P. falciparum *parasite is a field still in its infancy. In 2004, relative quantification was performed in this laboratory using a SILAC (stable-isotope labelling of amino acids in cell culture) -based method [11]. While a significant step forward for plasmodial proteomics, this study necessarily focused on proteins of high abundance to establish proof of principle. Since then, other significant studies have been undertaken to assess plasmodial proteins in a quantitative manner [14,15], but again, these studies determined only relative quantification.

In order to truly understand a biological system, we must aspire to absolute quantification of proteins across their entire dynamic range. By doing this, data from different studies and different laboratories can be more easily compared and proteomic, transcriptomic and metabolomic data can be more meaningfully correlated. One potential method for absolute protein quantification is the use of a heterologously expressed so-called QconCAT protein [16].

In this method, proteotypic tryptic peptides from proteins of interest, 'Q-peptides', are chosen. A number of these peptides are then expressed together from an artificial gene to produce a concatenated protein containing many different Q-peptides. Thus, when the QconCAT protein is digested using trypsin, peptides representing a number of proteins of interest are produced in equal molar amounts. If such QconCAT proteins are expressed, from *Escherichia coli*, in a labelled form using stable-isotope labelling, they can then be introduced, in a known amount, into biological samples prior to tryptic digestion and mass spectrometry. The resulting 'heavy' peaks on mass spectra, derived from the Q-peptides can be compared with the corresponding 'light' peaks, obtained concurrently from the native peptides, allowing accurate, absolute quantification. Producing a labelled QconCAT, rather than a labelled form of an entire native protein, allows several proteins to be efficiently quantified in the same experiment.

Here a QconCAT-based method, combined with SRM mass spectrometry, is described for the absolute quantification of proteins of low abundance in erythrocytic-stage *P. falciparum*.

## Methods

### *Plasmodium falciparum *culture

*Plasmodium falciparum *K1 parasites were grown in washed O+ erythrocytes in RPMI 1640 medium (Invitrogen, UK) as previously described [11,17]. The medium was supplemented with 0.5% bovine serum albumin, 5 μg.ml^-1 ^hypoxanthine and 50 μg.ml^-1 ^gentamicin sulphate (all Sigma Aldrich).

### Extraction of parasites

*Plasmodium falciparum *parasites were freed from infected erythrocytes by lysis in 0.05% saponin in phosphate-buffered saline (PBS) for 5 min. at 4°C. This suspension was subsequently centrifuged at 800*g *for 5 min. at 4°C. The resulting parasite pellets were washed in ice-cold PBS and spun at 1,000*g *for 5 min. This washing process was repeated at least twice more, until contaminating blood cell lysis products were no longer apparent in the PBS. The subsequent pellet was weighed and, if not being used immediately, snap-frozen in liquid nitrogen before storage at -80°C.

### Extraction of *Plasmodium falciparum *proteins

Proteins were extracted from *P. falciparum *samples using the freeze-thaw method previously described [5]. For every 100 μg of parasite pellet, 2.5 ml of de-ionized water and 5 μl of protease inhibitor cocktail (Sigma Protease Inhibitor Cocktail, P8340) was added and the pellet re-suspended. This suspension was frozen in liquid nitrogen for 5 min., thawed in a 37°C water bath and vortexed for 30 sec. at room temperature. This freeze-thaw cycle was subsequently repeated four times to ensure maximum protein extraction. The insoluble material was wholly removed by centrifugation three times at 16,000*g *for 20 min. at 4°C. The soluble proteins thus extracted were precipitated by the addition of five volumes of acetone to the solution followed by incubation at -20°C overnight. The resulting suspension was subsequently centrifuged at 12,000*g *for 20 min. at 4°C and the protein pellet air-dried.

### Design of PfQconCAT1

Twelve *P. falciparum *proteins were chosen to be represented in the PfQconCAT1 protein. These included six enzymes of folate metabolism: GTP cyclohydrolase I (GTPCH), pyruvoyltetrahydropterin synthase III (PTPS), hydroxymethyldihydropterin pyrophosphokinase-dihydropteroate synthase (HPPK-DHPS), dihydrofolate synthase-folylpolyglutamate synthase (DHFS-FPGS), dihydrofolate reductase-thymidylate synthase (DHFR-TS) and serine hydroxymethyltransferase (SHMT). All of these proteins are known to be of a low abundance [5,18]. In order to represent some proteins of higher abundance and thus provide a context for the abundance figures sought, the following proteins were also represented: adenosine deaminase, disulphide isomerase, eukaryotic initiation factor 5α (eIF5α), lactate dehydrogenase (LDH), plasmepsin I and pyruvate kinase. Each protein was represented by four peptides in the QconCAT protein. Two calibration peptides were also included (see Additional file 1 - Annotated amino acid sequence of PfQconCAT1).

For those folate pathway proteins identified previously by mass spectrometry in our laboratory (PTPS, HPPK-DHPS, DHFR-TS and SHMT), peptides were chosen which had previously been seen in the mass spectra [5]. For those proteins in which more than four different peptides had been seen, the four seen with highest intensity were chosen. For all other proteins, theoretical peptides were chosen using Peptide Predictor Version 2 software. This is a locally written derivative of the widely available Peptide Sieve software (Seattle Proteome Center). The software allows screening of peptides in proteins for high 'flyability' and low probability of missed cleavages in trypsin digestion (for example, ensuring that an acidic amino-acid residue is not present two places after a lysine or arginine residue). This program was first run using those proteins already identified in mass spectra. The predictions made by the software were found to be highly concordant with the visibility of peptides in experimental results.

In the QconCAT protein, no two peptides representing the same protein were placed consecutively. This was to ensure that any missed cleavages did not eliminate the ability for quantification of two peptides from the same protein. A process of trial and error was then used placing the 74 peptides in various configurations and assessing with Peptide Predictor to ensure that missed tryptic cleavages remained a low probability. A sacrificial N-terminus and His-tagged C-terminus (to facilitate purification) were then added and the sequence was sent to PolyQuant (http://www.polyquant.com) for codon optimization and gene synthesis. The amino-acid sequence is shown in annotated form (Additional file 1 - Annotated amino acid sequence of PfQconCAT1).

### Synthesis of labelled PfQconCAT1 protein

Rosetta™ 2 (DE3) pLysS cells were transformed with the sequence-verified, artificial *PFQconCAT1 *gene received from PolyQuant in the pET21 vector. These cells were grown overnight on LB agar and used to inoculate 500 ml of PA-5052 medium [19] supplemented with both ^13^C-labelled arginine and ^13^C-labelled lysine (0.2 mg.ml^-1 ^each). PA-5052 is made as follows: 50 mM Na_2_HPO_4_, 50 mM KH_2_PO_4_, 25 mM (NH_4_)_2_SO_4_, 0.5% (v/v) glycerol, 0.05% (w/v) glucose, 0.2% (w/v) α-lactose monohydrate, 2 mM MgSO_4_, 50 mM FeCl_3_, 20 μM CaCl_2_, 10 μM MnCl_2_, 10 μM ZnSO_4_, 2 μM CoCl_2_, 2 μM CuCl_2_, 2 μM NiCl_2_, 2 μM Na_2_MoO_4_, 2 μM Na_2_SeO_3_, 2 μM H_3_BO_3 _and 0.2 mg.ml^-1 ^each of the following amino acids: alanine, aspartate, glutamine, glycine, isoleucine, leucine, methionine, proline, serine, threonine, tryptophan, valine, phenylalanine, monosodium glutamate, asparagine monohydrate and histidine monochloride. The cells were then grown in this auto-induction medium over two nights at 37°C with agitation until the OD_600 _plateaued (usually an OD_600 _of between 6 and 12). The cells were then harvested by centrifugation for 20 min. at 4,000*g *at 4°C.

These cells were subsequently processed as described previously [20]. The cells were lysed using Bugbuster (Novagen) according to the manufacturer's guidelines. The resulting suspension was centrifuged at 16,000*g *for 20 min. at 4°C and the supernatant discarded. The pellet was re-suspended in the same amount of Bugbuster again by Pasteur pipetting. Lysozyme (250 ml of 10 mg.ml^-1 ^in Bugbuster) was added and mixed by gentle vortexing. This was incubated at room temperature for 5 min. followed by centrifuging at 15,000*g *for 15 min. at 4°C. The resulting supernatant was discarded. This pellet was re-suspended in 20 ml of a 1/10 dilution of Bugbuster in de-ionized water then centrifuged again at 15,000*g *for 15 min. at 4°C. This washing in 1/10 Bugbuster was repeated twice more to produce a pellet of washed inclusion bodies.

The inclusion bodies were re-suspended in 20 ml of column buffer (20 mM Na_2_HPO_4_/NaH_2_PO_4 _pH7.4, 500 mM NaCl, 10 mM imidazole, 6 M guanidinium chloride [all Sigma Aldrich]) and were subsequently centrifuged at 8,000 rpm for 5 min. at room temperature. The supernatant was transferred to a fresh tube and the pellet discarded. A 1 ml HiTrap Chelating HP column (GE Healthcare) was equilibrated with column buffer using an AKTA purification system. The previously prepared crude extract was then applied to the column at a flow rate of 0.25 ml.min.^-1 ^and the column was washed with wash buffer (20 mM Na_2_HPO_4_/NaH_2_PO_4 _pH7.4, 500 mM NaCl, 20 mM imidazole, 6 M guanidinium chloride) until no protein could be seen eluting from the column on the UV absorbance output. The bound protein was then eluted from the column by switching from wash buffer to elution buffer (20 mM Na_2_HPO_4_/NaH_2_PO_4 _pH7.4, 500 mM NaCl, 500 mM imidazole, 6 M guanidinium chloride) on a 20 min. linear gradient. The resulting solution was dialysed into 6 M urea overnight. Dialysing into lower concentrations was found to result in precipitation of the protein, which would subsequently prove difficult to re-solubilize. Quantification of the protein was performed using a 2D Quant kit (GE Healthcare).

### Selected reaction monitoring mass spectrometry analysis

Acetone-precipitated protein obtained from *P. falciparum *samples was trypsin-digested using an Amicon Ultra 0.5 ml 10 kDa molecular weight cut-off centrifugal filter unit (Millipore). The dried protein sample was first taken up in 15.5 μl digestion buffer (50 mM NH_4_HCO_3_, 20 mM CaCl_2_, 2 M Urea) and applied to the upper chamber of an Amicon filter. An appropriate, known amount of labelled PfQconCAT1 protein was added (1 pmol per 100 μg plasmodial extract) followed by 2.5 μl of 10 mM DTT and the whole incubated for 20 min. at room temperature. Subsequently, 2 μl of 30 mM iodoacetamide was added and incubated for a further 20 min. at room temperature. A further 200 μl of digestion buffer was then added and the column was spun at 14,000*g *for 15 min. The filtrate was then discarded and the filter chamber transferred to a fresh tube. Proteomics grade trypsin (0.1 mg.ml^-1^, Sigma Aldrich) in 50 mM NH_4_HCO_3 _was then added to give 1 μg trypsin for every 50 μg protein to be digested and the whole was incubated overnight at 37°C. Formic acid (1 μl) was added to stop the reaction followed by 200 μl of 50 mM NH_4_HCO_3_. The column was centrifuged at 14,000*g *for 15 min. and the filter was discarded. The filtrate was then dried in a vacuum centrifuge and re-suspended in 10% acetonitrile, 0.1% formic acid for analysis by mass spectrometry.

This analyte was loaded onto a Symmetry^® ^C18 HPLC trapping column of 20 mm length and an internal diameter (ID) of 180 μm (Waters) coupled to a BEH130 C18 HPLC analytical column of 15 cm length and an ID of 75 μm (Waters nanoACQUITY™ UPLC). The peptides were eluted from these columns using a 3-40% gradient of 100% acetonitrile with water (both containing 0.1% formic acid) at a flow rate of 300nl.min^-1^. The peptides were eluted into a Xevo TQMS (Waters) mass spectrometer (ionization through electrospray ionization and mass analysis through tandem quadrupoles) using an SRM transition list (see Additional file 2 - SRM Transition List). Data were acquired using MassLynx software (Waters).

### Tandem mass spectrometry analysis

Trypsin-digested analyte was loaded onto a Symmetry^® ^C18 HPLC trapping column of 20 mm length and an internal diameter (ID) of 180 μm (Waters) coupled to a BEH130 C18 HPLC analytical column of 15 cm length and an ID of 75 μm (Waters nanoACQUITY™ UPLC). The peptides were eluted from these columns using a 3-40% gradient of 100% acetonitrile with water (both containing 0.1% formic acid) at a flow rate of 300nl.min^-1^. The peptides were eluted into a Synapt G1 High Definition mass spectrometer (Waters; ionization through electrospray and mass analysis through quadrupole time of flight) run in MS^E ^mode. Data were acquired and analysed using MassLynx (v4.1) and Protein Lynx Global Server (v2,4) software packages respectively (Waters).

## Results

### Development of *Pf*QconCAT1 SRM transition list

Initially, an SRM transition list was produced for the PfQconCAT1 protein using Pinpoint software (Thermo Finnigan) to predict what fragment ions might be seen from the second round of MS (see Additional file 2 - SRM Transition List). This analysis also recognized the value of choosing singly charged fragment ions whose m/z was greater than that of their doubly charged parent ions, thus helping to minimize background intensity that might be generated from the latter during the SRM analyses. However, to be sure that useful fragment ions would indeed be produced experimentally, purified heterologously expressed *Pf*QconCAT1 was trypsin digested and run in MS^E ^mode on a Synapt G1 QToF mass spectrometer. The list of precursor and fragment ions seen (see Additional file 3 - Precursor and fragment ions found through trypsin digestion and Synapt QToF mass spectrometry of heterologously expressed ^13^C-labelled PfQconCAT1), which includes those derived from all of the targeted proteins other than plasmepsin I, is significantly different to that prepared using the predictive software (see Additional file 2 - SRM Transition List). Thus, approximately 77% of predicted fragment ions were not seen experimentally and 37% of ions seen experimentally were not predicted. This discordance between predictions of which theoretical peptides might be best chosen as proteotypic, particularly with respect to their detection in the mass spectrometer (selection of characteristic transitions) is notable. It illustrates the value, for future studies, of taking every effort to select peptides/transitions that have previously been experimentally demonstrated.

### Quantification of *Plasmodium falciparum *proteins

Trypsin-digested *P. falciparum *protein (2 μg) was loaded onto the Xevo TQ mass spectrometer and analysed using SRM as described above. Results from QconCAT-based quantification of proteins from two unsynchronized *P. falciparum *cultures are shown in Table 1.

**Table 1:** Quantification of *P. falciparum *proteins from two unsynchronized erythrocytic-stage cultures

Peptide	Fragment Ion Type	Calculated Native Level (Culture 1)	Peptide Average	Calculated Native Level (Culture 2)	Peptide Average	Average Between Samples
Adenosine Deaminase-1	y4	5.96	6.53 (0.23)	-	-	-
					
	y5	6.83		-		
					
	y6	6.79		-		

Disulphide Isomerase-1	y5	9.54	16.47 (2.83)	-	-	-
					
	y6	20.11		-		
					
	y7	19.76		-		

DHFR-TS-4	y7	0.68	0.31 (0.15)	0.24	0.68 (0.20)	0.50 (0.02)
					
	y8	0.05		1.09		
					
	y10	0.21		0.72		

eIF5α-3	y6	18.64	19.05 (1.90)	20.33	22.98 (2.13)	21.02 (1.39)
					
	y8	15.23		28.20		
					
	y9	23.27		20.41		

SHMT-4	y5	0.48	0.87 (0.31)	0.69	0.99 (0.29)	0.93 (0.04)
					
	y6	0.49		1.69		
					
	y7	1.63		0.58		

All figures are pmol per 100 μg of *P. falciparum *protein and numbers in brackets represent standard errors. Adenosine Deaminase-1 and Disulphide Isomerase-1 were only detected in experiment 1.

**Figure 1:**
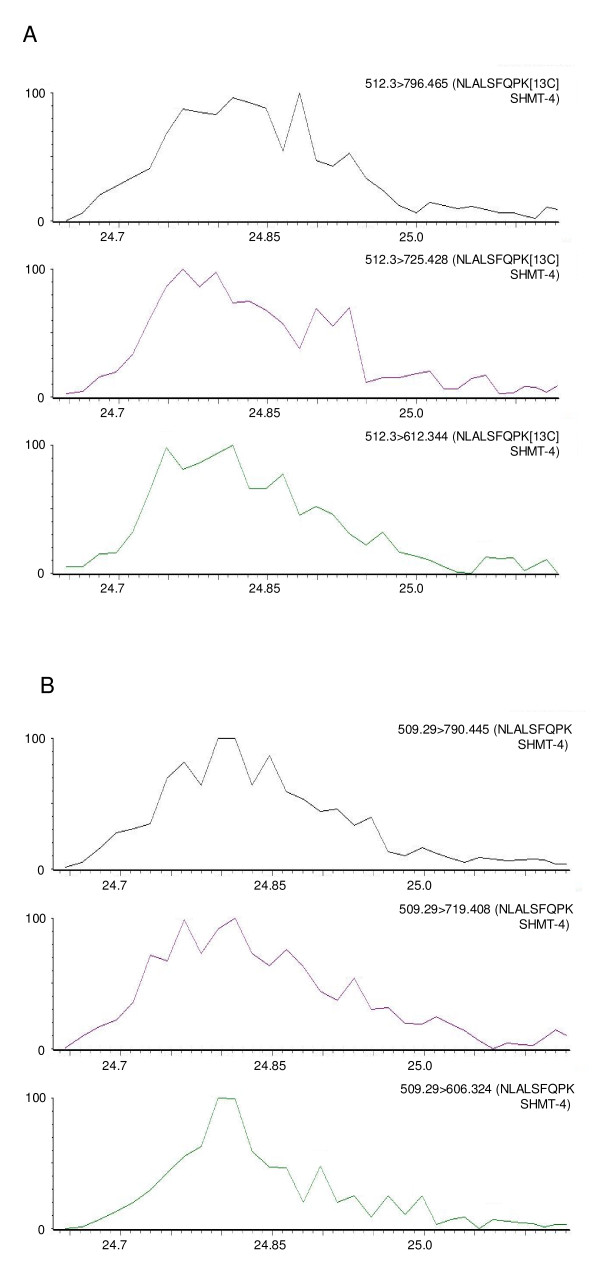
**Extracted ion chromatograms of transitions derived from peptide SHMT-4**. The ordinate represents the peak intensity (normalised to 100%) and the abscissa the time within the elution gradient (minutes) that this intensity was detected. Panel A, transitions corresponding to the labelled version of this peptide; Panel B, transitions corresponding to the unlabelled version of this peptide. The relative area under each of the traces and the known amount of the standard (labelled) peptide added were used to calculate the amount of the unlabelled peptide and thus the amount of the equivalent protein present in the sample analyzed.

In order for quantification to be performed, both labelled and unlabelled forms of the peptides must be seen in mass spectra of the samples. This was the case for three of the peptides in both experiments (representing DHFR-TS, eIF5α and SHMT) and for two additional peptides in one of the experiments (representing adenosine deaminase and disulphide isomerase). Extracted ion chromatograms of three transitions from both labelled and unlabelled forms of a peptide chosen as diagnostic for SHMT (SHMT-4) are shown in Figure 1. Additional file 4 - Extracted ion chromatograms from experimentally observed peptides - shows all of the transitions observed in the two independent experiments from which the data presented in Table 1 were derived. The fact that some peptides were seen in one experiment and not in the other may reflect differences in ion suppression effects in different complex mixtures of peptides. For those peptides seen in both experiments, a high degree of reproducibility was seen with similar abundance figures obtained for the same peptides in each experiment (standard error varied between 4.0% and 6.7% of the average).

Overall, abundance figures for the six peptides seen reflect what would be expected, with enzymes of the folate pathway of substantially lower abundance than the other enzymes. Furthermore, the two enzymes identified from the folate pathway are the two hypothesized to be the two most abundant within this pathway based on previous transcriptomic data [11]. In that study, the SHMT transcript was also found to be more abundant than that of DHFR-TS, a result also reflected in these proteomic results. Another notable outcome from these experiments is that in using a mass spectrometric method as sensitive as SRM, proteins of very low abundance can be seen without any fractionation of such a complex proteome. Fractionation using methods such as OFFGEL™ isoelectric fractionation have recently been demonstrated to have the potential to mine deep into the proteome, with four of the six folate enzymes being identified using this method [5]. Using the SRM method described in this paper in combination with OFFGEL™, a fifth folate enzyme (GTPCH) has also been identified (data not shown) showing the potential of this combined approach to mine and quantify a large portion of the *P. falciparum *proteome.

## Conclusions

Combining the use of a heavy-labelled, custom-designed QconCAT protein with SRM mass spectrometry has allowed the absolute quantification of even low abundance proteins from the folate metabolic pathway within unfractionated cell extracts. This method can easily be targeted to any plasmodial protein, for the first time offering a direct route to absolute quantification of malarial proteins. Furthermore, with the ability to represent a dozen or more proteins of interest in one labelled artificial protein, there is great potential for this approach to provide a relatively high-throughput application to absolutely quantify plasmodial proteomics. Such quantification will be a prerequisite for successful application of systems biology techniques to the in-depth analysis of parasite biochemistry.

## List of abbreviations

DHFR-TS: dihydrofolate reductase-thymidylate synthase; DHFS-FPGS: dihydrofolate synthase-folylpolyglutamate synthase; eIF5α: eukaryotic initiation factor 5α; GTPCH: GTP cyclohydrolase I; HPPK-DHPS: hydroxymethyldihydropterin pyrophosphokinase-dihydropteroate synthase; LDH: lactate dehydrogenase; MS: mass spectrometry; PTPS: pyruvoyltetrahydropterin synthase III; SHMT: serine hydroxymethyltransferase; SRM: selected reaction monitoring.

## Competing interests

The authors declare that they have no competing interests.

## Authors' contributions

PMS carried out all the experimental work and drafted the manuscript. JEH and PFGS conceived the study and revised the manuscript. All authors were involved in experimental design, data interpretation and analysis and have read and approved the final manuscript.

## Supplementary Material

Additional file 1 **Annotated amino acid sequence of PfQconCAT1**. Click here for file

Additional file 2 **SRM Transition List**. Click here for file

Additional file 3 **Precursor and fragment ions found through trypsin digestion and Synapt QToF mass spectrometry of heterologously expressed **^**13**^**C-labelled PfQconCAT1**. Click here for file

Additional file 4 **Extracted ion chromatograms from experimentally observed peptides**. Click here for file
