# Supplementary material for: A mass spectrometric strategy for absolute quantification of Plasmodium falciparum proteins of low abundance
Source: Malar J. 2011 Oct 25;10:315. doi: 10.1186/1475-2875-10-315 (PMC3219587; doi:10.1186/1475-2875-10-315)
Supplement: Additional file 1 — Annotated amino acid sequence of PfQconCAT1. [file 1475-2875-10-315-S1.PDF]

Additional file1 - Annotated amino acid sequence of PfQconCAT1.

MGTK*MAGVR*AGVIR*NGHVMLK*NLALSFQPK*NLFDNGK*ESLLNHNA

|--Sacrificial N-terminus--|-------e--------|---------S--------|--------e-------|-------DI------

IINFFK*ETVDNVNDMPNSK*IVLVGSGMIGGVMATLIVQK*TFVNDPLSML

-----------|-----------DT-------------|-----------------L-------------------|------------HD-----

VVIK*FFEGLDVVK*VEDLIVLLGK*EYFNETK*IPLPYEGER*NLDLVTNGTD

--------|--------PI--------|--------DT--------|-------G-----|-------DI--------|-------------S-----

NHLIVVDLR*EGVVLMEFR*GGVNDNEEGFFSAR*ELIHLVNEVLNK*DIFD

------------------|---------AD-------|------------Q---------------|-------------DF--------|--AD-

NIR*HIHYIEISVSESPTQK*DTEGNLDEVAK*MNLWAVQK*SNDIEEQIINIS

------|------------P----------------|---------e------------|--------AD------|----------G--------

K*NLGDVVLFDIVK*DIFYLPSLNER*GVNDNEEGFFSAR*EDLVVIDEK*HV

---|-----------L------------|---------DF--------|------------Q------------|--------G--------|----

IIGFSIENSHDR*AILLTDELQQK*YEQDIQNNISYFDK*TNIAVLNLGTNDR*

-------PI-------------|-----------S---------|-------------PK-----------|----------HD-------------|

AGQIILLDDGNLK*NIINLIK*DVNAHIVGAHGNK*DDGYVIDFSILK*AHIVGI

----------PK------------|----DT----|------------L--------------|---------P-----------|------e---

DIFTGR*ELFDLLEK*IIGLGGVLDTSR*LQNVVVMGR*EGDLFSFDLMNSL

------------|------S---------|---------L-----------|------DT---------|------------PK-----------

GNQNR*ETLHGHNYNVSLK*NIVTNIGDDK*TITYASYK*LTNYDNLVYDIK*I

------------|-----------P--------------|--------PK--------|------G-------|------------HD--------|

VVGNSFVDVVLK*NDEHDMSDILHK*VEMNYVSGTVSGFFSK*LDHHFILPI

--------DI---------------|----------DF-----------|--------------PI---------------|-----------P---

YSDVLK*GLNEAVALLEYK*TIHIAGTNGK*ASLGLTEFPGLAFQSNEGR*DI

-------------|-----------AD----------|------DF----------|--------------DI---------------------|---

VTIANLSFPYK*NAVLILETALHLVEK*IAAALEHHHHHH

--------PI-----------|------------HD-------------|-C-terminal His-tag.

The annotated amino-acid sequence of the QconCAT protein used in these experiments, showing the order of peptides and the protein that each represents. The black stars indicate the expected digestion points of trypsin (after K and R residues). Abbreviations: adenosine deaminase (AD); dihydrofolate reductase-thymidylate synthase (DT); dihydrofolate synthase-folylpolyglutamate synthase (DF); disulphide isomerase (DI); eukaryotic initiation factor 5α (e); GTP cyclohydrolase I (G); hydroxymethyldihydropterin pyrophosphokinase-dihydropteroate synthase (HD); lactose dehydrogenase (L); plasmepsin I (PI); pyruvate kinase (PK); pyruvoyltetrahydropterin synthase III (P); serine hydroxymethyltransferase (S); QconCAT calibration peptide (Q).
