# Supplementary material for: A mass spectrometric strategy for absolute quantification of Plasmodium falciparum proteins of low abundance
Source: Malar J. 2011 Oct 25;10:315. doi: 10.1186/1475-2875-10-315 (PMC3219587; doi:10.1186/1475-2875-10-315)
Supplement: Additional file 2 — SRM Transition List. [file 1475-2875-10-315-S2.PDF]

**Additional file 2 - SRM Transition List.**

| Peptide | Precursor Mass to Charge | Fragment Mass to Charge | Precursor Charge | Fragment Charge | Ion Type |
|---|---|---|---|---|---|
| AGQIILLDDGNLK[$^{13}$C]<br>(Pyruvate Kinase-2) | 688.39697 | 780.4186 | 2 | 1 | y7 |
| | 688.39697 | 893.5027 | 2 | 1 | y8 |
| | 688.39697 | 1006.587 | 2 | 1 | y9 |
| | 688.39697 | 1119.671 | 2 | 1 | y10 |
| AHIVGIDIFTGR[$^{13}$C]<br>(eIF5α-2) | 652.87402 | 714.387 | 2 | 1 | y6 |
| | 652.87402 | 827.471 | 2 | 1 | y7 |
| | 652.87402 | 884.4925 | 2 | 1 | y8 |
| | 652.87402 | 983.5609 | 2 | 1 | y9 |
| AILLTDELQQK[$^{13}$C]<br>(SHMT-1) | 639.37299 | 651.376 | 2 | 1 | y5 |
| | 639.37299 | 766.403 | 2 | 1 | y6 |
| | 639.37299 | 867.4507 | 2 | 1 | y7 |
| | 639.37299 | 980.5347 | 2 | 1 | y8 |
| ASLGLTEFPGLAFQSNEG<br>R[$^{13}$C]<br>(Disulph. Isom.-4) | 1000.512 | 1027.526 | 2 | 1 | y9 |
| | 1000.512 | 1084.547 | 2 | 1 | y10 |
| | 1000.512 | 1181.6 | 2 | 1 | y11 |
| | 1000.512 | 1328.668 | 2 | 1 | y12 |
| | 1000.512 | 1457.711 | 2 | 1 | y13 |
| DDGYVIDFSILK[$^{13}$C]<br>(PTPS-1) | 695.86298 | 728.4278 | 2 | 1 | y6 |
| | 695.86298 | 841.5118 | 2 | 1 | y7 |
| | 695.86298 | 940.5803 | 2 | 1 | y8 |
| | 695.86298 | 1103.644 | 2 | 1 | y9 |
| DIFDNIR[$^{13}$C]<br>(Adenosine Deam.-4) | 449.73901 | 523.2924 | 2 | 1 | y4 |
| DIFYLPSLNER[$^{13}$C]<br>(DHFS-FPGS-3) | 686.86298 | 721.3928 | 2 | 1 | y6 |
| | 686.86298 | 834.4769 | 2 | 1 | y7 |
| | 686.86298 | 997.5402 | 2 | 1 | y8 |
| DIVTIANLSFPYK[$^{13}$C]<br>(Plasmepsin I-3) | 743.91498 | 760.4329 | 2 | 1 | y6 |
| | 743.91498 | 874.4758 | 2 | 1 | y7 |
| | 743.91498 | 945.5129 | 2 | 1 | y8 |
| | 743.91498 | 1058.597 | 2 | 1 | y9 |
| | 743.91498 | 1159.645 | 2 | 1 | y10 |
| DTEGNLDEVAK[$^{13}$C]<br>(eIF5α-3) | 598.78998 | 680.3914 | 2 | 1 | y6 |
| | 598.78998 | 794.4343 | 2 | 1 | y7 |
| | 598.78998 | 851.4557 | 2 | 1 | y8 |
| DVNAHIVGAHGNK[$^{13}$C]<br>(LDH-4) | 669.354 | 688.3826 | 2 | 1 | y7 |
| | 669.354 | 801.4666 | 2 | 1 | y8 |
| | 669.354 | 938.5255 | 2 | 1 | y9 |
| | 669.354 | 1009.563 | 2 | 1 | y10 |

| | | | | | |
|---|---|---|---|---|---|
| EDLVVIDEK[$^{13}$C] (GTPCH-1) | 533.29199 | 609.3542 | 2 | 1 | y5 |
| | 533.29199 | 708.4227 | 2 | 1 | y6 |
| EGDLFSFDLMNSLGNQNR [$^{13}$C] (Pyruvate Kinase-1) | 1031.983 | 1039.504 | 2 | 1 | y9 |
| | 1031.983 | 1152.588 | 2 | 1 | y10 |
| | 1031.983 | 1267.615 | 2 | 1 | y11 |
| | 1031.983 | 1414.683 | 2 | 1 | y12 |
| EGDLFSFDLM[Oxid]NSLG NQNR[$^{13}$C] (Pyruvate Kinase-1) | 1039.98 | 1055.499 | 2 | 1 | y9 |
| | 1039.98 | 1168.583 | 2 | 1 | y10 |
| | 1039.98 | 1283.61 | 2 | 1 | y11 |
| | 1039.98 | 1430.678 | 2 | 1 | y12 |
| EGVVLMEFR[$^{13}$C] (Adenosine Deam.-1) | 543.29102 | 588.2899 | 2 | 1 | y4 |
| | 543.29102 | 701.374 | 2 | 1 | y5 |
| | 543.29102 | 800.4424 | 2 | 1 | y6 |
| EGVVLM[Oxid]EFR[$^{13}$C] (Adenosine Deam.-1) | 551.28802 | 604.2848 | 2 | 1 | y4 |
| | 551.28802 | 717.3689 | 2 | 1 | y5 |
| | 551.28802 | 816.4373 | 2 | 1 | y6 |
| ELFDLLEK[$^{13}$C] (SHMT-3) | 506.78601 | 508.343 | 2 | 1 | y4 |
| | 506.78601 | 623.3699 | 2 | 1 | y5 |
| ELIHLVNEVLNK[$^{13}$C] (DHFS-FPGS-4) | 713.92102 | 722.4132 | 2 | 1 | y6 |
| | 713.92102 | 821.4816 | 2 | 1 | y7 |
| | 713.92102 | 934.5657 | 2 | 1 | y8 |
| | 713.92102 | 1071.625 | 2 | 1 | y9 |
| ESLLNHNAIINFFK[$^{13}$C] (Disulph. Isom.-3) | 833.45599 | 858.5173 | 2 | 1 | y7 |
| | 833.45599 | 972.5602 | 2 | 1 | y8 |
| | 833.45599 | 1109.619 | 2 | 1 | y9 |
| | 833.45599 | 1223.662 | 2 | 1 | y10 |
| | 833.45599 | 1336.746 | 2 | 1 | y11 |
| ETLHGHNYNVSLK[$^{13}$C] (PTPS-2) | 759.39301 | 843.4659 | 2 | 1 | y7 |
| | 759.39301 | 980.5248 | 2 | 1 | y8 |
| | 759.39301 | 1037.546 | 2 | 1 | y9 |
| | 759.39301 | 1174.605 | 2 | 1 | y10 |
| ETVDNVNDMPNSK[$^{13}$C] (DHFR-TS-4) | 734.83698 | 811.3703 | 2 | 1 | y7 |
| | 734.83698 | 910.4387 | 2 | 1 | y8 |
| | 734.83698 | 1024.482 | 2 | 1 | y9 |
| | 734.83698 | 1139.509 | 2 | 1 | y10 |
| ETVDNVNDM[Oxid]PNSK [$^{13}$C] (DHFR-TS-4) | 742.83502 | 827.3652 | 2 | 1 | y7 |
| | 742.83502 | 926.4337 | 2 | 1 | y8 |
| | 742.83502 | 1040.477 | 2 | 1 | y9 |
| | 742.83502 | 1155.504 | 2 | 1 | y10 |
| EYFNETK[$^{13}$C] (GTPCH-4) | 468.72299 | 497.2654 | 2 | 1 | y4 |
| FFEGLDVVK[$^{13}$C] (Plasmepsin I-4) | 530.29401 | 579.3801 | 2 | 1 | y5 |
| | 530.29401 | 636.4016 | 2 | 1 | y6 |

| | | | | | |
|---|---|---|---|---|---|
| GGVNDNEEGFFSAR[13C] (QCal-1) | 752.841 | 819.4084 | 2 | 1 | y7 |
| | 752.841 | 948.451 | 2 | 1 | y8 |
| | 752.841 | 1062.494 | 2 | 1 | y9 |
| | 752.841 | 1177.521 | 2 | 1 | y10 |
| | 752.841 | 1291.564 | 2 | 1 | y11 |
| GLNEAVALLEYK[13C] (Adenosine Deam.-2) | 663.37299 | 671.4063 | 2 | 1 | y5 |
| | 663.37299 | 742.4434 | 2 | 1 | y6 |
| | 663.37299 | 841.5118 | 2 | 1 | y7 |
| | 663.37299 | 912.549 | 2 | 1 | y8 |
| | 663.37299 | 1041.592 | 2 | 1 | y9 |
| GVNDNEEGFFSAR[13C] (QCal-2) | 724.33002 | 819.4084 | 2 | 1 | y7 |
| | 724.33002 | 948.451 | 2 | 1 | y8 |
| | 724.33002 | 1062.494 | 2 | 1 | y9 |
| | 724.33002 | 1177.521 | 2 | 1 | y10 |
| HIHYIEISVSESPTQK[13C] (PTPS-4) | 937.49103 | 968.4984 | 2 | 1 | y9 |
| | 937.49103 | 1081.582 | 2 | 1 | y10 |
| | 937.49103 | 1210.625 | 2 | 1 | y11 |
| | 937.49103 | 1323.709 | 2 | 1 | y12 |
| | 937.49103 | 1486.772 | 2 | 1 | y13 |
| HVIIGFSIENSHDR[13C] (Plasmepsin I-1) | 815.42499 | 876.4258 | 2 | 1 | y7 |
| | 815.42499 | 963.4579 | 2 | 1 | y8 |
| | 815.42499 | 1110.526 | 2 | 1 | y9 |
| | 815.42499 | 1167.548 | 2 | 1 | y10 |
| | 815.42499 | 1280.632 | 2 | 1 | y11 |
| IAAALEHHHHHH (C-terminal His-tag) | 705.34998 | 841.3708 | 2 | 1 | y6 |
| | 705.34998 | 970.4133 | 2 | 1 | y7 |
| | 705.34998 | 1083.497 | 2 | 1 | y8 |
| | 705.34998 | 1154.535 | 2 | 1 | y9 |
| IIGLGGVLDTSR[13C] (LDH-3) | 603.85999 | 696.3975 | 2 | 1 | y6 |
| | 603.85999 | 753.419 | 2 | 1 | y7 |
| | 603.85999 | 810.4404 | 2 | 1 | y8 |
| | 603.85999 | 923.5245 | 2 | 1 | y9 |
| IPLPYEGER[13C] (Disulph. Isom.-1) | 540.29401 | 659.3084 | 2 | 1 | y5 |
| | 540.29401 | 756.3611 | 2 | 1 | y6 |
| IVLVGSGMIGGVMATLIVQK[13C] (LDH-2) | 996.58801 | 1008.621 | 2 | 1 | y9 |
| | 996.58801 | 1065.643 | 2 | 1 | y10 |
| | 996.58801 | 1122.664 | 2 | 1 | y11 |
| | 996.58801 | 1235.748 | 2 | 1 | y12 |
| | 996.58801 | 1366.789 | 2 | 1 | y13 |
| | 996.58801 | 1423.81 | 2 | 1 | y14 |

| | | | | | |
|---|---|---|---|---|---|
| IVLVGSGM[Oxid]IGGVM[ Oxid]ATLIVQK[$^{13}$C] (LDH-2) | 1012.583 | 1024.616 | 2 | 1 | y9 |
| | 1012.583 | 1081.637 | 2 | 1 | y10 |
| | 1012.583 | 1138.659 | 2 | 1 | y11 |
| | 1012.583 | 1251.743 | 2 | 1 | y12 |
| | 1012.583 | 1398.778 | 2 | 1 | y13 |
| | 1012.583 | 1455.8 | 2 | 1 | y14 |
| IVVGNSFVDVVLK[$^{13}$C] (Disulph. Isom.-2) | 697.92102 | 825.5169 | 2 | 1 | y7 |
| | 697.92102 | 912.549 | 2 | 1 | y8 |
| | 697.92102 | 1026.592 | 2 | 1 | y9 |
| | 697.92102 | 1083.613 | 2 | 1 | y10 |
| LDHHFILPIYSDVLK[$^{13}$C] (PTPS-3) | 908.508 | 940.5439 | 2 | 1 | y8 |
| | 908.508 | 1053.628 | 2 | 1 | y9 |
| | 908.508 | 1166.712 | 2 | 1 | y10 |
| | 908.508 | 1313.78 | 2 | 1 | y11 |
| | 908.508 | 1450.839 | 2 | 1 | y12 |
| LQNVVVMGR[$^{13}$C] (DHFR-TS-3) | 511.29901 | 567.3372 | 2 | 1 | y5 |
| | 511.29901 | 666.4056 | 2 | 1 | y6 |
| LQNVVVM[Oxid]GR[$^{13}$C] (DHFR-TS-3) | 519.29602 | 583.3321 | 2 | 1 | y5 |
| | 519.29602 | 682.4005 | 2 | 1 | y6 |
| LTNYDNLVYDIK[$^{13}$C] (HPPK-DHPS-1) | 738.88702 | 756.459 | 2 | 1 | y6 |
| | 738.88702 | 870.502 | 2 | 1 | y7 |
| | 738.88702 | 985.5289 | 2 | 1 | y8 |
| | 738.88702 | 1148.592 | 2 | 1 | y9 |
| MNLWAVQK[$^{13}$C] (Adenosine Deam.-3) | 498.27499 | 637.3757 | 2 | 1 | y5 |
| M[Oxid]NLWAVQK[$^{13}$C] (Adenosine Deam.-3) | 506.272 | 637.3757 | 2 | 1 | y5 |
| NAVLILETALHLVEK[$^{13}$C] (HPPK-DHPS-4) | 835.00299 | 916.5551 | 2 | 1 | y8 |
| | 835.00299 | 1045.598 | 2 | 1 | y9 |
| | 835.00299 | 1158.682 | 2 | 1 | y10 |
| | 835.00299 | 1271.766 | 2 | 1 | y11 |
| | 835.00299 | 1384.85 | 2 | 1 | y12 |
| NDEHDMSDILHK[$^{13}$C] (DHFS-FPGS-1) | 730.33197 | 849.4587 | 2 | 1 | y7 |
| | 730.33197 | 964.4857 | 2 | 1 | y8 |
| | 730.33197 | 1101.545 | 2 | 1 | y9 |
| NDEHDM[Oxid]SDILHK [$^{13}$C] (DHFS-FPGS-1) | 738.32898 | 865.4537 | 2 | 1 | y7 |
| | 738.32898 | 980.4806 | 2 | 1 | y8 |
| | 738.32898 | 1117.54 | 2 | 1 | y9 |
| NGHVMLK[$^{13}$C] (eIF5α-1) | 402.728 | 496.3252 | 2 | 1 | y4 |
| NGHVM[Oxid]LK[$^{13}$C] (eIF5α-1) | 410.72501 | 413.2517 | 2 | 1 | y3 |
| | 410.72501 | 512.3201 | 2 | 1 | y4 |
| NIINLIK[$^{13}$C] (DHFR-TS-2) | 417.28101 | 493.3433 | 2 | 1 | y4 |

| | | | | | |
|---|---|---|---|---|---|
| NIVTNIGDDK[$^{13}$C] (Pyruvate Kinase-3) | 547.79199 | 553.2917 | 2 | 1 | y5 |
| | 547.79199 | 667.3346 | 2 | 1 | y6 |
| | 547.79199 | 768.3823 | 2 | 1 | y7 |
| NLALSFQPK[$^{13}$C] (SHMT-4) | 512.29999 | 525.312 | 2 | 1 | y4 |
| | 512.29999 | 612.3441 | 2 | 1 | y5 |
| | 512.29999 | 725.4281 | 2 | 1 | y6 |
| NLDLVTNGTDNHLIVVDL R[$^{13}$C] (SHMT-2) | 1064.078 | 1084.656 | 2 | 1 | y9 |
| | 1064.078 | 1199.683 | 2 | 1 | y10 |
| | 1064.078 | 1300.731 | 2 | 1 | y11 |
| | 1064.078 | 1357.752 | 2 | 1 | y12 |
| | 1064.078 | 1471.795 | 2 | 1 | y13 |
| NLFDNGK[$^{13}$C] (eIF5α-4) | 407.21301 | 439.2236 | 2 | 1 | y4 |
| NLGDVVLFDIVK[$^{13}$C] (LDH-1) | 669.39203 | 740.4642 | 2 | 1 | y6 |
| | 669.39203 | 839.5326 | 2 | 1 | y7 |
| | 669.39203 | 938.601 | 2 | 1 | y8 |
| | 669.39203 | 1053.628 | 2 | 1 | y9 |
| SNDIEEQIINISK[$^{13}$C] (GTPCH-2) | 754.89801 | 821.518 | 2 | 1 | y7 |
| | 754.89801 | 950.5605 | 2 | 1 | y8 |
| | 754.89801 | 1079.603 | 2 | 1 | y9 |
| | 754.89801 | 1192.687 | 2 | 1 | y10 |
| TFVNDPLSMLVVIK[$^{13}$C] (HPPK-DHPS-3) | 791.45398 | 795.5097 | 2 | 1 | y7 |
| | 791.45398 | 908.5938 | 2 | 1 | y8 |
| | 791.45398 | 1005.647 | 2 | 1 | y9 |
| | 791.45398 | 1120.673 | 2 | 1 | y10 |
| | 791.45398 | 1234.716 | 2 | 1 | y11 |
| TFVNDPLSM[Oxid]LVVIK [$^{13}$C] (HPPK-DHPS-3) | 799.45099 | 811.5046 | 2 | 1 | y7 |
| | 799.45099 | 924.5887 | 2 | 1 | y8 |
| | 799.45099 | 1021.641 | 2 | 1 | y9 |
| | 799.45099 | 1136.668 | 2 | 1 | y10 |
| | 799.45099 | 1250.711 | 2 | 1 | y11 |
| TIHIAGTNGK[$^{13}$C] (DHFS-FPGS-2) | 509.29199 | 553.3029 | 2 | 1 | y6 |
| | 509.29199 | 666.387 | 2 | 1 | y7 |
| TITYASYK[$^{13}$C] (GTPCH-3) | 476.75699 | 637.3281 | 2 | 1 | y5 |
| TNIAVLNLGTNDR[$^{13}$C] (HPPK-DHPS-2) | 703.888 | 795.4044 | 2 | 1 | y7 |
| | 703.888 | 908.4885 | 2 | 1 | y8 |
| | 703.888 | 1007.557 | 2 | 1 | y9 |
| | 703.888 | 1078.594 | 2 | 1 | y10 |
| VEDLIVLLGK[$^{13}$C] (DHFR-TS-1)) | 552.85199 | 648.4743 | 2 | 1 | y6 |
| | 552.85199 | 761.5584 | 2 | 1 | y7 |
| VEMNYVSGTVSGFFSK [$^{13}$C] (Plasmepsin I-2) | 879.42902 | 935.4922 | 2 | 1 | y9 |
| | 879.42902 | 1022.524 | 2 | 1 | y10 |
| | 879.42902 | 1121.593 | 2 | 1 | y11 |
| | 879.42902 | 1284.656 | 2 | 1 | y12 |
| | 879.42902 | 1398.699 | 2 | 1 | y13 |

| | | | | | |
|---|---|---|---|---|---|
| VEM[Oxid]NYVSGTVSGFF SK[13C] (Plasmepsin I-2) | 887.42603 | 935.4922 | 2 | 1 | y9 |
| | 887.42603 | 1022.524 | 2 | 1 | y10 |
| | 887.42603 | 1121.593 | 2 | 1 | y11 |
| | 887.42603 | 1284.656 | 2 | 1 | y12 |
| | 887.42603 | 1398.699 | 2 | 1 | y13 |
| YEQDIQNNISYFDK[13C] (Pyruvate Kinase-4) | 891.91699 | 892.45 | 2 | 1 | y7 |
| | 891.91699 | 1006.493 | 2 | 1 | y8 |
| | 891.91699 | 1134.552 | 2 | 1 | y9 |
| | 891.91699 | 1247.635 | 2 | 1 | y10 |
| | 891.91699 | 1362.662 | 2 | 1 | y11 |

Proteotypic peptides included in PfQconCAT1 and the transitions selected for use in their identification.
