# Supplementary material for: A mass spectrometric strategy for absolute quantification of Plasmodium falciparum proteins of low abundance
Source: Malar J. 2011 Oct 25;10:315. doi: 10.1186/1475-2875-10-315 (PMC3219587; doi:10.1186/1475-2875-10-315)
Supplement: Additional file 3 — Precursor and fragment ions found through trypsin digestion and Synapt QToF mass spectrometry of heterologously expressed 13C-labelled PfQconCAT1. [file 1475-2875-10-315-S3.PDF]

Additional file 3 - Precursor and fragment ions found through trypsin digestion and Synapt QToF mass spectrometry of heterologously expressed ^{13}C-labelled PfQconCAT1.

The fragment ions predicted by Pinpoint software (Thermo Finnigan) are highlighted in grey. Peptide numbering is as shown in Additional file 2. All detected fragment ions had a charge of +1.

Peptide	Precursor Mass to Charge	Fragment Mass to Charge	Precursor Charge	Fragment Ion Type
AGQIILLDDGNLK[^{13}C] (Pyruvate Kinase-2)	688.39697	780.4186	2	y7
	688.39697	893.5027	2	y8
	688.39697	1006.587	2	y9
AILLTDELQQK[^{13}C] (SHMT-1)	639.37299	867.4507	2	y7
	639.37299	980.5347	2	y8
	639.37299	1093.619	2	y9
DTEGNLDEVAK[^{13}C] (eIF5α-3)	598.78998	680.3914	2	y6
	598.78998	851.4557	2	y8
	598.78998	980.4984	2	y9
DVNAHIVGAHGNK[^{13}C] (LDH-4)	446.57199	589.3141	3	y6
	446.57199	688.3826	3	y7
	446.57199	801.4666	3	y8
EDLVVIDEK[^{13}C] (GTPCH-1)	533.29199	609.3542	2	y5
	533.29199	708.4227	2	y6
	533.29199	821.5068	2	y7
EGVVLMEFR[^{13}C] (Adenosine Deam.-1)	543.29102	588.2899	2	y4
	543.29102	701.374	2	y5
	543.29102	800.4424	2	y6
EGVVLM[Oxid]EFR[^{13}C] (Adenosine Deam.-1)	551.28802	604.2848	2	y4
	551.28802	717.3689	2	y5
	551.28802	816.4373	2	y6
ELFDLLEK[^{13}C] (SHMT-3)	506.78601	395.2589	2	y3
	506.78601	623.3699	2	y5
	506.78601	770.4384	2	y6
ETLHGHNYNVSLK[^{13}C] (PTPS-2)	506.59799	843.4659	3	y7
	506.59799	980.5248	3	y8
	506.59799	1037.546	3	y9
ETVDNVNDMPNSK[^{13}C] (DHFR-TS-4)	734.83698	811.3703	2	y7
	734.83698	910.4387	2	y8
	734.83698	1139.509	2	y10
ETVDNVNDM[Oxid]PNSK [^{13}C] (DHFR-TS-4)	742.83502	827.3652	2	y7
	742.83502	926.4337	2	y8
	742.83502	1155.504	2	y10

EYFNETK[13C] (GTPCH-4)	468.72299	497.2654	2	y4
	468.72299	644.3339	2	y5
	468.72299	807.3972	2	y6
GGVNDNEEGFFSAR[13C] (QCal-1)	752.84100	819.4084	2	y7
	752.84100	948.451	2	y8
	752.84100	1062.494	2	y9
GVNDNEEGFFSAR[13C] (QCal-2)	724.33002	819.4084	2	y7
	724.33002	948.451	2	y8
	724.33002	1062.494	2	y9
IIGLGGVLDTSR[13C] (LDH-3)	603.85999	753.419	2	y7
	603.85999	810.4404	2	y8
	603.85999	980.546	2	y10
IPLPYEGER[13C] (Disulph. Isom.-1)	540.29401	659.3084	2	y5
	540.29401	756.3611	2	y6
	540.29401	869.4452	2	y7
LQNVVVMGR[13C] (DHFR-TS-3)	511.29901	567.3372	2	y5
	511.29901	666.4056	2	y6
	511.29901	780.4485	2	y7
LQNVVVM[Oxid]GR[13C] (DHFR-TS-3)	519.29602	583.3321	2	y5
	519.29602	682.4005	2	y6
	519.29602	796.4434	2	y7
M[Oxid]NLWAVQK[13C] (Adenosine Deam.-3)	506.27200	637.3757	2	y5
	506.27200	750.4597	2	y6
	506.27200	864.5027	2	y7
NIINLIK[13C] (DHFR-TS-2)	417.28101	493.3433	2	y4
	417.28101	606.4274	2	y5
	417.28101	719.5114	2	y6
NIVTNIGDDK[13C] (Pyruvate Kinase.-3)	547.79199	667.3346	2	y6
	547.79199	768.3823	2	y7
	547.79199	867.4507	2	y8
NLALSFQPK[13C] (SHMT-4)	512.29999	612.3441	2	y5
	512.29999	725.4281	2	y6
	512.29999	796.4652	2	y7
TIHIAGTNGK[13C] (DHFS-FPGS-2)	339.86401	553.3029	3	y6
	339.86401	666.387	3	y7
	339.86401	803.4459	3	y8
	509.29199	553.3029	2	y6
	509.29199	666.387	2	y7
	509.29199	803.4459	2	y8
TITYASYK[13C] (GTPCH-3)	476.75699	403.2276	2	y3
	476.75699	637.3281	2	y5
	476.75699	738.3757	2	y6
TNIAVLNLGTNDR[13C] (HPPK-DHPS-2)	703.88800	795.4044	2	y7
	703.88800	908.4885	2	y8
	703.88800	1007.557	2	y9
