# Supplementary material for: A mass spectrometric strategy for absolute quantification of Plasmodium falciparum proteins of low abundance
Source: Malar J. 2011 Oct 25;10:315. doi: 10.1186/1475-2875-10-315 (PMC3219587; doi:10.1186/1475-2875-10-315)
Supplement: Additional file 4 — Extracted ion chromatograms from experimentally observed peptides. [file 1475-2875-10-315-S4.PDF]

Additional file 4 - Extracted ion chromatograms from experimentally observed peptides.

Experiment 1:

Adenosine Deaminase-1:

Labelled:

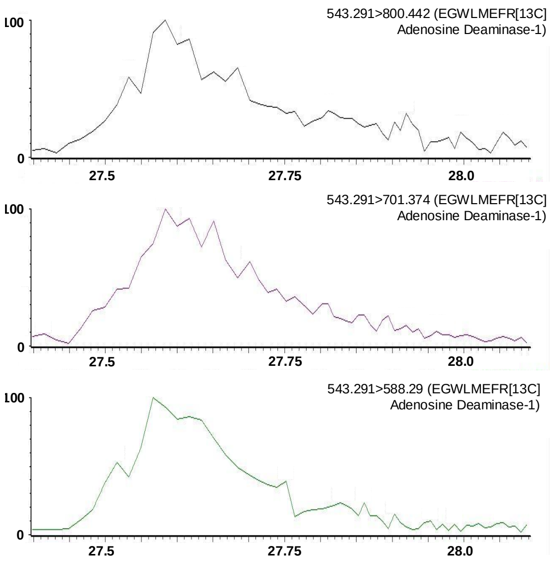

Unlabelled:

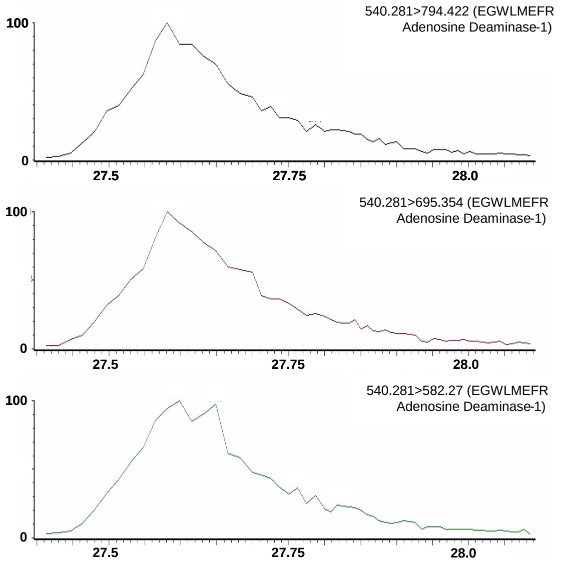

Disulphide Isomerase-1:

Labelled:

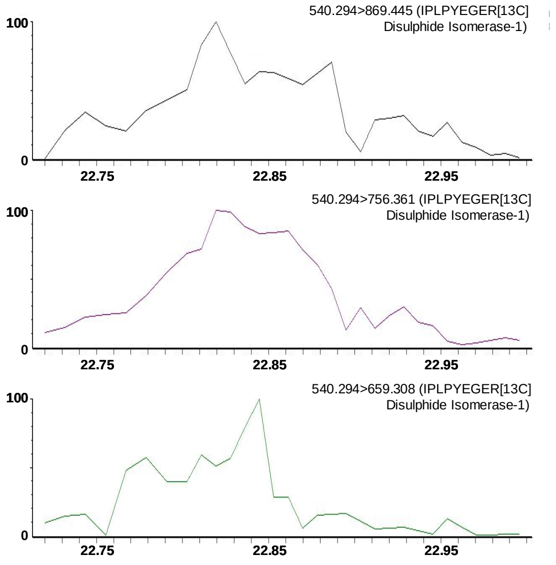

Unlabelled:

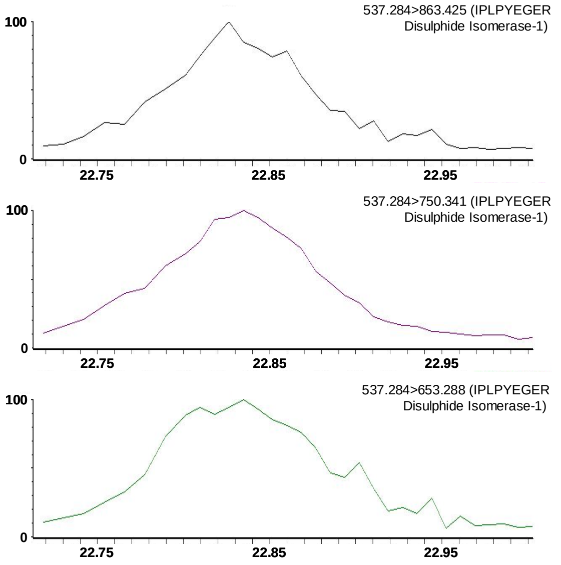

DHFR-TS-4:

Labelled:

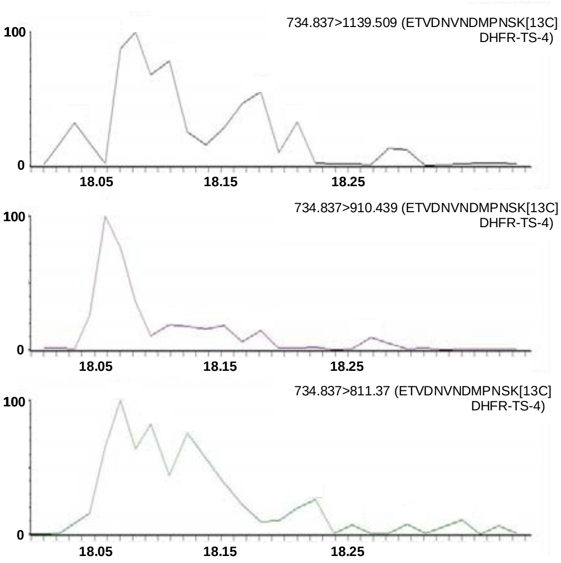

Unlabelled:

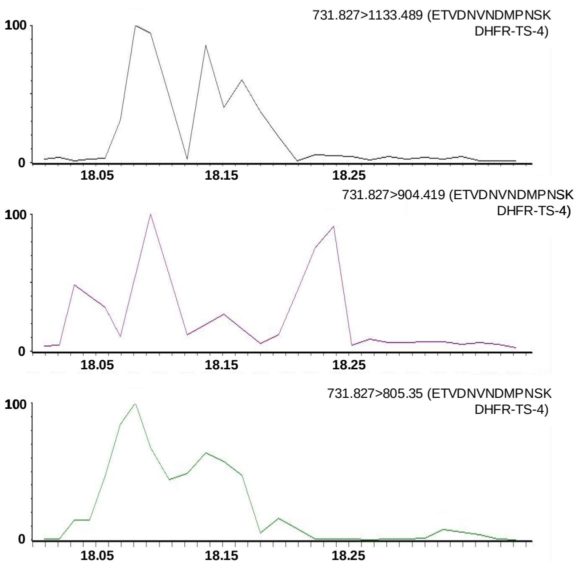

eIF5α-3:

Labelled:

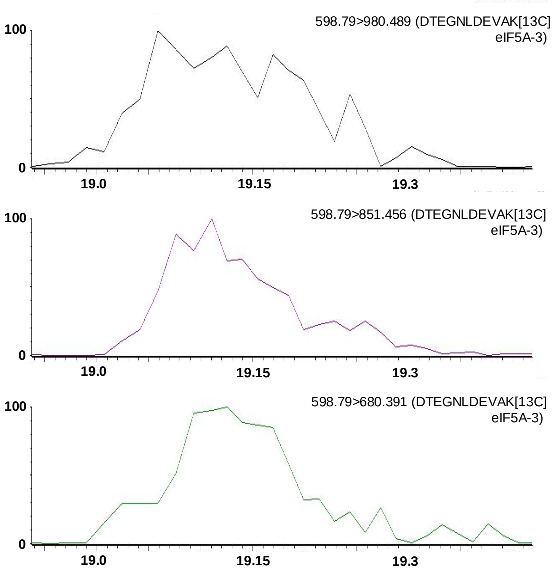

Unlabelled:

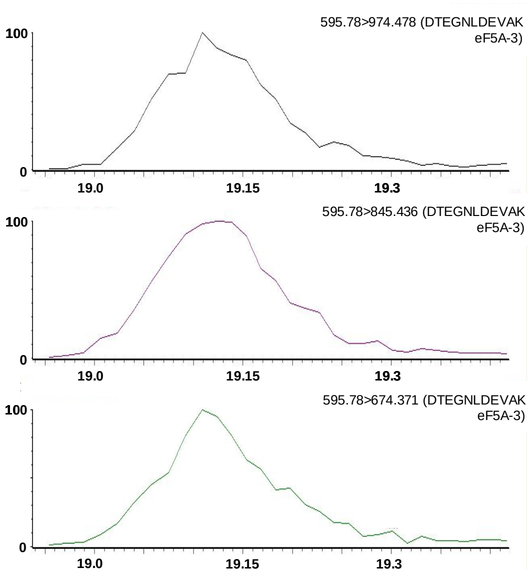

SHMT-4:

Labelled:

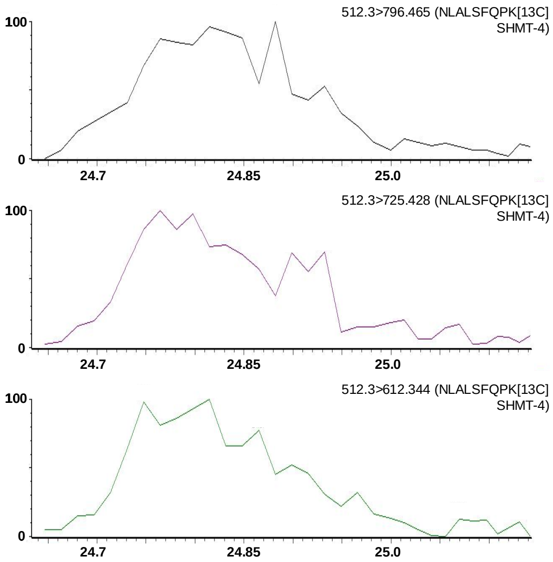

Unlabelled:

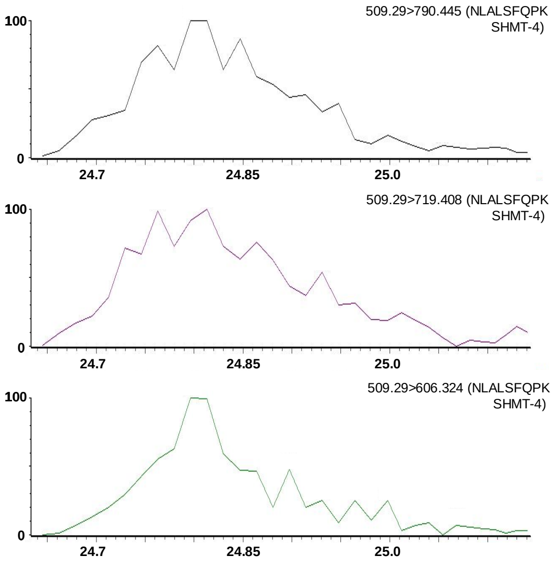

Experiment 2:

DHFR-TS-4:

Labelled:

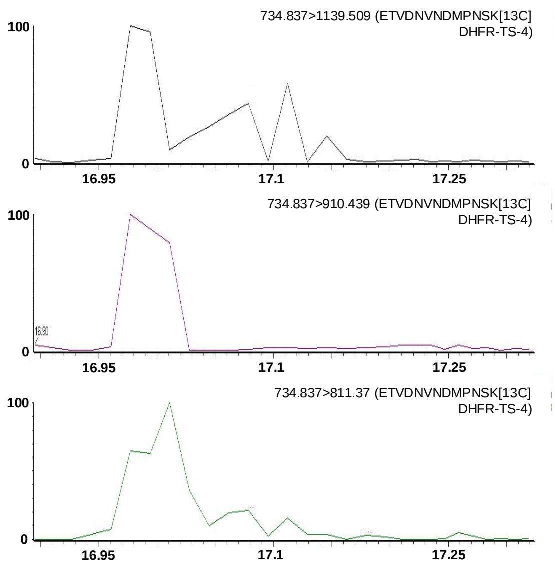

Unlabelled:

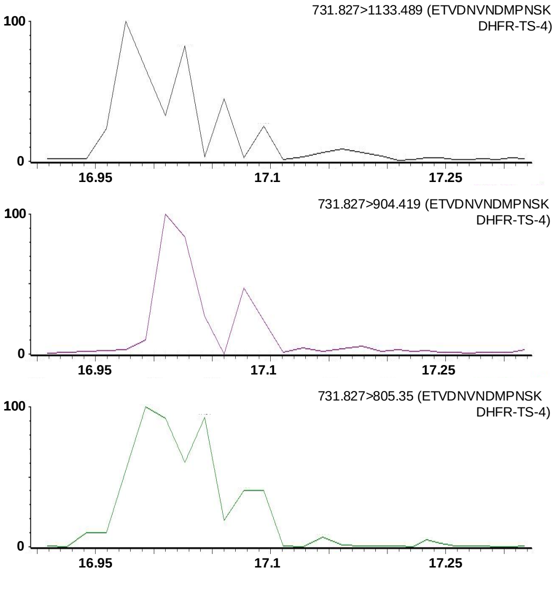

eIF5α-3:

Labelled: **Unlabelled:**

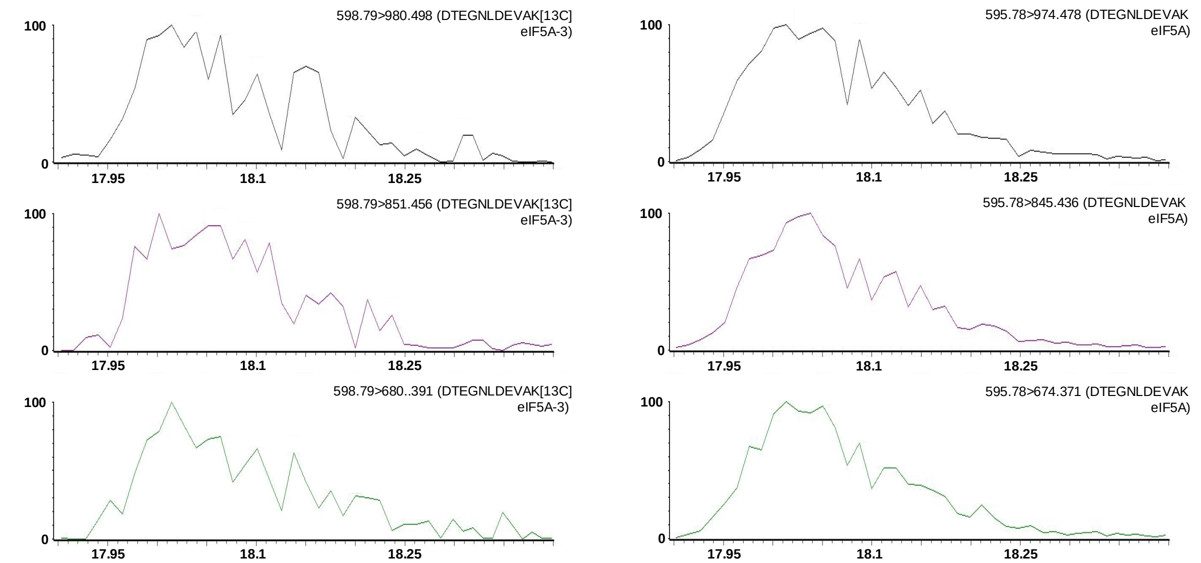

SHMT-4:

Labelled: **Unlabelled:**

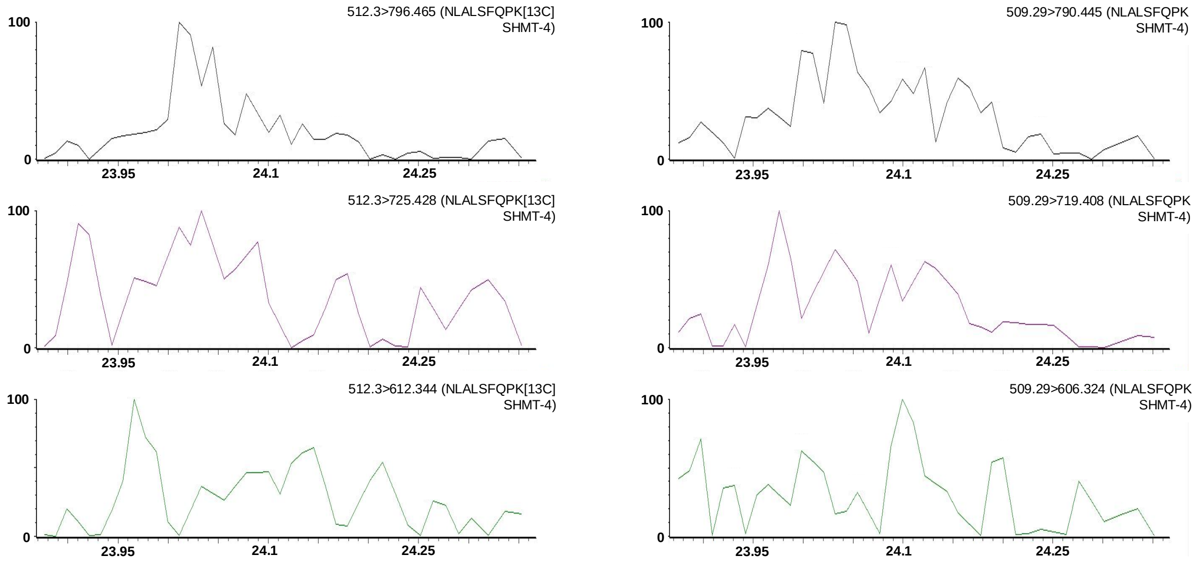

The ordinate represents the peak intensity (normalised to 100%) and the abscissa the indicated time (min) within the elution gradient that this intensity was detected. Traces are presented in the order shown in Table 1.
